# A case of IgG4-positive ligneous conjunctivitis mistaken for a conjunctival mass

**DOI:** 10.1186/s13000-023-01366-0

**Published:** 2023-06-29

**Authors:** Jing Li, Rui Liu, Tingting Ren, Hong Zhang, Jianmin Ma

**Affiliations:** 1 Beijing Institute of Ophthalmology, Beijing Tongren Eye Center, Beijing Tongren Hospital, Capital Medical University, Beijing, 100730 China; 2 Pathology, Beijing Tongren Hospital, Capital Medical University, Beijing, 100730 China

**Keywords:** Ligneous conjunctivitis, IgG4-related disease, Diagnosis, Treatment

## Abstract

**Background:**

Ligneous conjunctivitis (LC) is a rare inflammatory lesion of the conjunctiva with an unknown etiology. It is easily confused with conjunctiva lymphoma or other diseases in clinical diagnosis, and the lesion is very difficult to treat.

**Case presentation:**

We presented a 41-year-old female patient presented with bilateral conjunctival masses for more than six months. The patient had no contributory history of ocular trauma, family history of tumor and drug allergy. Taking the patient’s clinical and pathological features together, we considered this was a case of IgG4 + LC. Completely surgical resection combined with local glucocorticoid treatment might be effective.

**Conclusions:**

This is a very rare case report of immunoglobulin G4 positive LC with only one published case in literature. The typical manifestations of LC are with the appearance of a hard, fibrin-rich, woody pseudomembranous lesion. A large number of lymphocyte and plasma cell are infiltrated in the pathological tissue. Inflammation of LC may cause immune abnormalities, resulting in IgG4 increasing.

## Introduction

Ligneous conjunctivitis (LC) is a rare, chronic, pseudomembranous form of conjunctivitis of unknown etiology that is more common in infancy and childhood [1]. Its main feature is the appearance of a hard, fibrin-rich, woody pseudomembranous lesion on the conjunctiva. Ligneous lesions are most common in the eyes but can also occur in the throat, vocal cords, nasopharynx, and other parts of the body [2]. Immunoglobulin G4 (IgG4)–related disease (IgG4-RD) is an immune-related fibro-inflammatory condition of unknown cause, characterized by increased serum IgG4 levels (>135 mg/dl), extensive infiltration of lymphocytes and plasma cells into diseased tissues, and a high count of IgG4^+^ plasma cells. According to Japanese studies, the incidence of IgG4-RD is 0.28–1.08/100,000, and that of IgG4-related ophthalmic disease (IgG4-ROD) accounts for 4–34% of total IgG4-RD cases [3]. IgG4-ROD mainly involves the lacrimal gland, extraocular muscles and trigeminal nerve; the optic nerve, conjunctiva, sclera, uvea, and choroid are rarely involved [4–8]. Despite the rarity of conjunctival involvement in IgG4-ROD [6–8], this paper reports a another very rare case of LC with strong positive expression of IgG4 in histopathological results with only one published case in literature.

## Case report

**Fig. 1:**
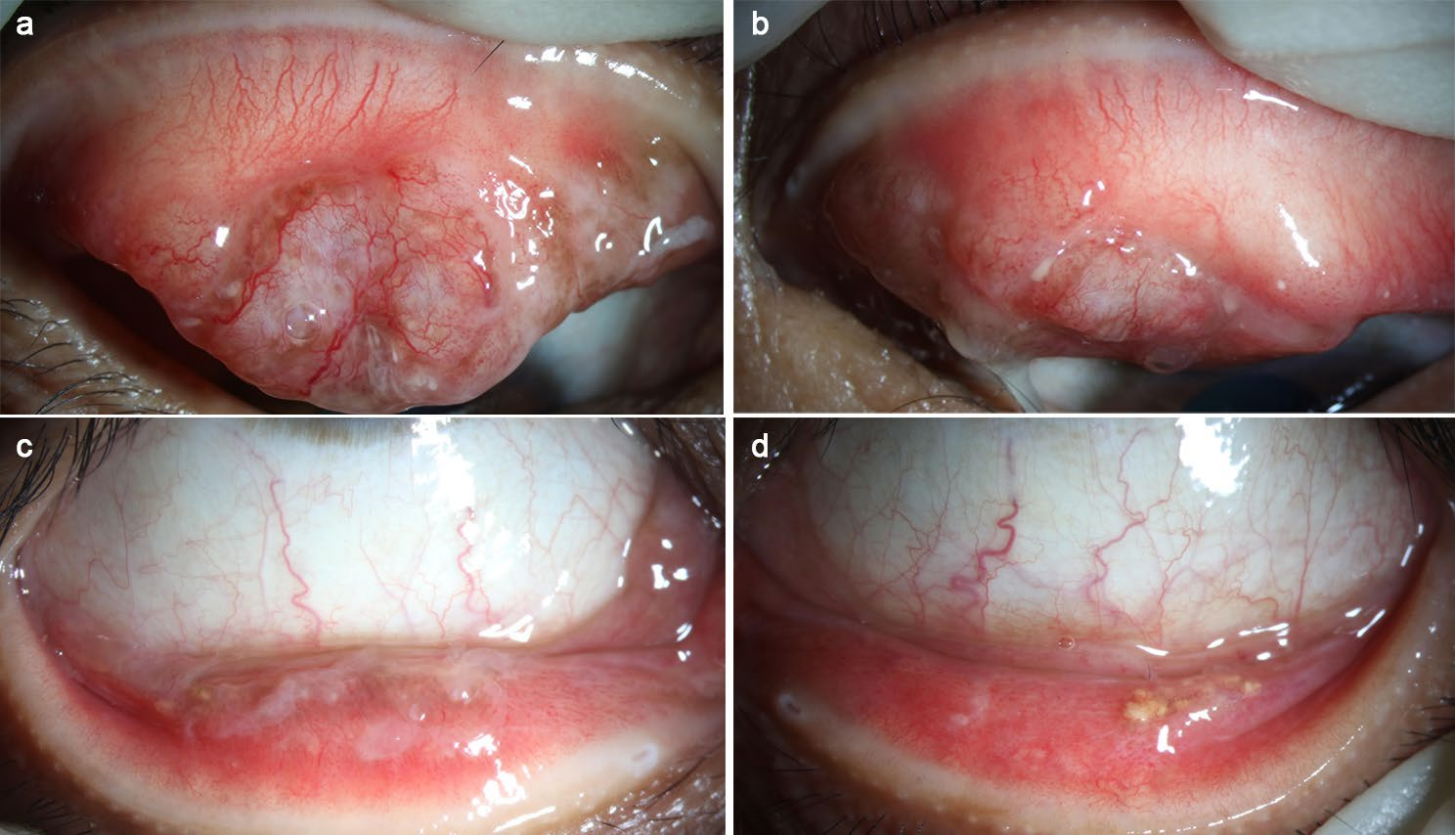
Appearance of the patient’s conjunctival lesions. (**A**) The mass on the conjunctival surface of the upper right eyelid with telangiectasia and brown lesions on the surface, and covered by a pseudomembrane; (**B**) Pseudomembranes and deposits of yellowish-white lipid-like substances on the conjunctival surface of the right lower eyelid; (**C**) The conjunctiva of the left upper eyelid was covered with mucus and pseudomembranes and the surface vessels were hyperplasic; (**D**) The conjunctival surface of the lower left eyelid was red, with yellowish-white lipid-like deposits on the surface

**Fig. 2:**
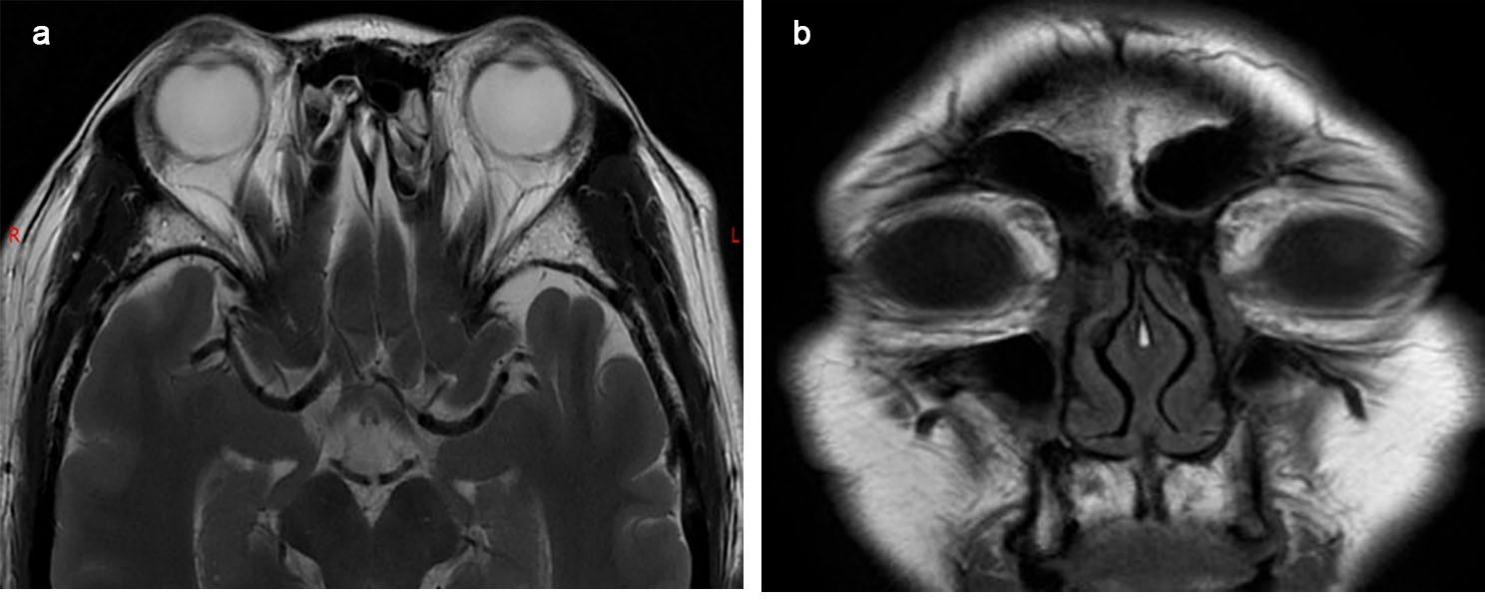
MRI images of the orbit. (**A**) Transverse orbital T2-weighted image (T2WI) showing slight thickening of the soft tissue of both eyelids. (**B**) Enhanced orbital scan showing slight thickening of the soft tissues of both eyelids and no obvious abnormality in the bilateral lacrimal glands

A 41-year-old female patient presented with bilateral conjunctival masses for more than six months, and the previous treatment with tobramycin eye drops was not effective. The patient had no contributory history of ocular trauma, family history of tumor and drug allergy. The mass on the conjunctival surface of the upper right eyelid was obvious, with telangiectasia and brown lesions on the surface, and was covered by a pseudomembrane (Fig. 1A). We observed pseudomembranes and deposits of yellowish-white lipid-like substances on the conjunctival surface of the lower eyelid (Fig. 1B). The conjunctiva of the left upper eyelid was covered with mucus and pseudomembranes, the conjunctival mass was prominent, and the surface vessels were hyperplasic (Fig. 1C). The conjunctival surface of the lower left eyelid was red, with yellowish-white lipid-like deposits on the surface (Fig. 1D). Eye movement in both eyes was normal: visual acuity—right eye, 1.0; left eye, 1.0; and intraocular pressure—right eye, 13 mmHg; left eye, 14 mmHg. We saw no abnormalities in the anterior and posterior segments of the eyes. Orbital magnetic resonance imaging (MRI) examination showed slight thickening of both eyelids and no abnormalities in the lacrimal glands, extraocular muscles, or nerves (Fig. 2). The preoperative diagnosis was a conjunctival mass in both eyes.

### Pathological findings

**Fig. 3:**
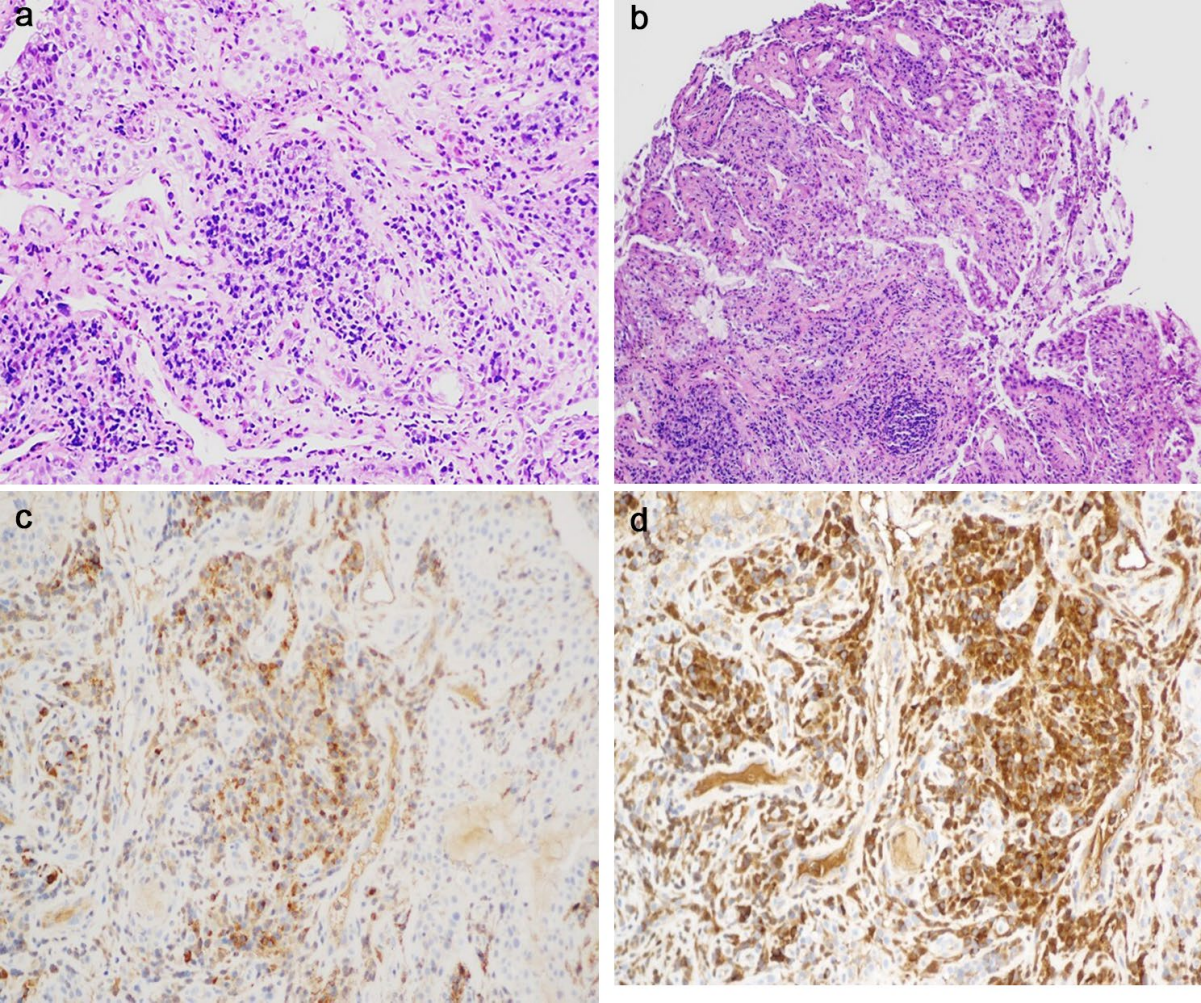
Pathological results and immunohistochemical staining of conjunctival lesions. (**A**) Neutrophils, lymphocytes and plasma cells infiltration and interstitial fibrosis in the lesion (×200). (**B**) Papillary hyperplasia of the tegular epithelium (×100). (**C**) Infiltration of IgG4 + plasma cells (×200). (**D**) IgG + plasma cell infiltration (×200)

The patient was worried that the lesion might be malignant and requested surgery to clarify its nature. Therefore, we performed conjunctival mass resection on both eyes at the same time and removed as much diseased tissue as possible. Histopathological results showed severe chronic inflammation of the polypoid mucosa of the conjunctival mucosa, infiltration by comprised predominantly of neutrophils, lymphocytes and plasma cells, fibrous-tissue hyperplasia, non-keratinized squamous epithelium or column epithelial papillary hyperplasia covering the fibrotic mucosa, and a great many visible inflammatory exudates in the lamina propria (Fig. 3). Immunohistochemical staining results were positive for 34ßE12, cytokeratin, IgG, IgG4, and P63 and negative for Congo Red. IgG4^+^ cell count was approximately 100/HPF, IgG^+^ cell count was approximately 200/HPF, and the IgG4/IgG ratio was > 40%. Taking the patient’s clinical and pathological features together, we consider this is a case of IgG4^+^ LC. After surgery, the patient received tobramycin and dexamethasone eye drops at localized points in both eyes for 14 days. In the 10 months’ follow-up, the disease had not recurred in the patient’s eyes.

## Discussion

LC is a disease with an estimated incidence of 16 cases per 1,000,000 people, and consanguineous marriage may be a predisposing factor for it [9]. It might be inherited in an autosomal-recessive manner, and its etiology might be related to local inflammation, plasminogen deficiency (type 1), plasminogen abnormality (type 2), tranexamic acid exposure, ocular insults such as trauma or infections and viral infection [10–12]. LC is a chronic disease, involving a wide range of age groups and prone to recurrence. Diagnosis mainly depends on typical clinical manifestations (woody pseudomembranous lesions) and pathological manifestations [12, 13].

Cases of LC complicated with IgG4-RD are extremely rare, and only one has been reported so far [13]. Because infiltration of lymphocytes and plasma cells is a common manifestation of LC and IgG4-RD, some scientists have proposed that LC is a type of IgG4-RD lesion and that such infiltration could play a key role in the disease’s occurrence [13]. In this paper, we report a rare case of LC with IgG4-RD. However, it is worth noting that IgG4-RD can involve any organ of the body, most commonly the pancreas, followed by the parotid gland, bile ducts, liver, lung, and lymph nodes [3]. Binocular conjunctival lesions can occur, but no evidence of IgG4-RD involvement has been found in other parts of the eye or other organs of the body. The typical pathological manifestations of LC and IgG4-RD are both lymphocyte and plasma cell infiltration. The lacrimal gland is the most commonly involved site of ocular IgG4-RD. In this case, there were no lesions in the lacrimal gland, extraocular muscle or other sites of the body except conjunctiva. Therefore, we speculate that inflammation of LC may cause immune abnormalities, resulting in IgG4 increasing. Persistent inflammation and elevated IgG4 levels may lead to more severe IgG4-RD, which will eventually involve the orbit and the whole body without intervention. Whether this disease belongs to the category of IgG4-RD is doubtful; more cases should be reported to clarify this matter.

LC is very difficult to treat. Tissue plasminogen activator, allogenic serum plasminogen, antibiotics, heparin, cyclosporine, and other drugs have been applied. The therapeutic effect of a single drug is usually poor; the combined application of multiple drugs has some therapeutic effect [14–17]. Studies have reported that the pseudomembrane does not respond to antibiotic or steroid treatment [18]. However, IgG4-RD is an immune-related fibro-inflammatory disease of unknown etiology, and there is no unified treatment plan. Glucocorticoids are the first-line treatment for IgG4-RD, and most of them have good therapeutic effects. For IgG4-RD involving only conjunctival tissue, when conjunctival lesions are wide-ranging, surgical resection combined with local drug therapy could be a better treatment choice.

The first reported case of LC with IgG4-RD was still recurrent after multiple treatments involving large-scale excision, cryotherapy, local triamcinolone injection, anti-proliferation soak of mitomycin C, and amniotic membrane transplantation; treatment required two years [13]. In the case reviewed in this paper, a relatively thorough surgical resection combined with the postoperative application of glucocorticoid ophthalmic fluid achieved a relatively ideal effect. However, we have followed up on this patient for only 6 months; assessment of the long-term effects requires further observation.

In summary, this is the second case report of ligneous conjunctivitis complicated with IgG4-RD. The main feature is conjunctivitis with the appearance of a hard, fibrin-rich, woody pseudomembranous lesion. When the drug treatment effect is poor, completely surgical resection combined with local glucocorticoid treatment might be effective. Conjunctival inflammation of LC may cause immune abnormalities, resulting in IgG4 increasing. The relationship between LC and IgG4-RD needs to be supported by more cases. Therefore, comprehensive and detailed eye and body examinations, as well as comprehensive laboratory, imaging, and histopathological examinations, are necessary.

## Data Availability

Not applicable.

## Abbreviations

LC: Ligneous conjunctivitis
IgG4-RD: Immunoglobulin G4 related disease
IgG4-ROD: IgG4-related ophthalmic disease
MRI: Magnetic resonance imaging
IHC: Immunohistochemical
HPF: Per high-powered field
